# Comparison of del Nido Cardioplegia with Blood Cardioplegia in Coronary Artery Bypass Grafting Combined with Mitral Valve Replacement

**DOI:** 10.21470/1678-9741-2018-0152

**Published:** 2018

**Authors:** Ali Aycan Kavala, Saygin Turkyilmaz

**Affiliations:** 1 Department of Cardiovascular Surgery, Bakirkoy Dr. Sadi Konuk Training and Research Hospital, Istanbul, Turkey.

**Keywords:** Mitral Valve/Surgery, Coronary Artery Bypass, Heart Arrest, Induced, Cardioplegic Solutions, Treatment Outcome

## Abstract

**Objective:**

To compare del Nido cardioplegia (DNC) with blood cardioplegia (BC) in coronary artery bypass grafting (CABG) combined with mitral valve replacement.

**Methods:**

A 3-year single-center retrospective cohort study was carried out. Subjects who underwent CABG (up to triple bypass) combined with mitral valve replacement were divided into DNC and BC groups. Each group had thirty subjects.

**Results:**

Both groups demonstrated similar baseline characteristics, including age, gender, cardiac/non-cardiac comorbidity, and preoperative echocardiographic parameters. Compared with the BC group, the DNC group demonstrated significantly lower cardioplegia volume (BC = 1130.00±194.1 mL, DNC = 884.33±156.8 mL, *P*=0.001), cardiopulmonary bypass time (DNC = 110.90±12.52 min, BC = 121.70±13.57 min, *P*=0.002), aortic clamp time (DNC = 91.37±11.58 min, BC = 101.37±13.87 min, *P*=0.004), and need for intraoperative defibrillation (DNC = 6 events, BC = 21 events, *P*=0.001). Postoperative creatine kinase-MB levels and troponin levels were significantly lower in the DNC group than in the BC group. Postoperative haemoglobin and haematocrit levels were significantly higher in the DNC group than in the BC group. The intubation period (hours) in intensive care unit (ICU) was significantly small in the BC group (DNC = 8.13±12.21, BC = 6.82±1.57, *P*=0.037); however, ICU stay, total hospital stay, and postoperative complication rates were not significantly different between them. At pre-discharge echocardiography, the DNC group demonstrated significantly higher ejection fraction rates than the BC group (47.79±5.50 and 45.72±5.86, respectively, *P*=0.005).

**Conclusion:**

DNC presented better intraoperative and postoperative parameters and it is an effective and safe alternative to BC for CABG combined with mitral valve replacement.

**Table:** 

Abbreviations, acronyms & symbols				
BC	= Blood cardioplegia		Htc	= Haematocrit
CABG	= Coronary artery bypass grafting		ICU	= Intensive care unit
CK-MB	= Creatine kinase-MB		NCSS	= Number Cruncher Statistical System
CPB	= Cardiopulmonary bypass		NYHA	= New York Heart Association
DNC	= Del Nido cardioplegia		RBCs	= Red blood cells
EF	= Ejection fraction		SD	= Standard deviation
FFP	= Fresh frozen plasma		TIA	= Transient ischemic attack
Hgb	= Haemoglobin			

## INTRODUCTION

Open cardiac procedures mostly require cardiopulmonary bypass (CPB) to maintain the blood supply during surgery^[1]^. Cardioplegia is a state of cardiac arrest and cardiac protection induced by the infusion of cardioplegia solution into the myocardium and involves the cessation of myocardial contractions. During cardioplegia, ischaemia occurs in myocardial tissue^[2]^. After cross-clamp removal and reperfusion is initiated, a degree of ischaemia/reperfusion injury occurs, which is responsible for most of the complications of cardiac surgery^[3]^.

Several cardioplegia solutions exist with different compositions. However, there is no standard for the optimal composition and delivery technique^[4]^. Blood cardioplegia (BC), which is the mixture of the subject's oxygenated blood (80%) and a crystalloid solution (20%), is the most widely used cardioplegia type^[1]^. The del Nido solution was formulated by researchers from the University of Pittsburgh (Pittsburgh, PA, USA) in the early 1990s^[5]^. As a calcium-free, hyperkalemic, modified depolarizing solution, it was specifically formulated for paediatric cardiac surgery^[6]^. The del Nido solution contains a base solution of Plasma-Lyte and a crystalloid component. The use of solutions has started to increase in recent years for adults. A recent meta-analysis demonstrated significant advantages of the del Nido solution over BC in adults for several parameters^[1]^. However, the data regarding the use of del Nido solution in combined coronary artery bypass grafting (CABG) and mitral valve replacement are limited. The aim of the present study is to compare the del Nido cardioplegia (DNC) with BC in CABG combined with mitral valve replacement surgery.

## METHODS

A 3-year retrospective cohort study was performed in a single tertiary academic center, Bakirkoy Dr. Sadi Konuk Education and Research Hospital, between December 2014 and December 2017. The study protocol was approved by the same hospital's ethics board.

Subjects in all age groups who underwent combined CABG (up to triple bypass) and mitral stenosis surgery were included in the study. Exclusion criteria were as follows:

- Previous cardiac surgery (open)
- Aortic insufficiency
- Concomitant procedures (*e.g*., aortic annulus enlargement, ascending aorta replacement, more than single-valve surgery)
- Tricuspid annuloplasty
- Subjects who underwent 4 or more bypass grafts performed during CABG surgery

The following demographic data and preoperative parameters were included: the subject's age, gender, New York Heart Association (NYHA) functional status, smoking and alcohol consumption status, body mass index, and comorbid diseases (hypertension, dyslipidaemia, diabetes mellitus, chronic obstructive pulmonary disease, and renal insufficiency). The preoperative previous stroke status, preoperative cardiac rhythm (atrial fibrillation, sinus rhythm, etc.), and preoperative echocardiogram parameters (ejection fraction [EF], valve area, valve gradient, presence/absence of atrial thrombus, and valve insufficiency) were also noted. For the study period, subjects who met the inclusion criteria for the DNC group were compared with the same number of subjects who had previously received BC by the same surgeon and met the inclusion criteria as well.

### Composition of del Nido and Blood Cardioplegia Solutions

Del Nido solution is composed of mixed blood and Plasma-Lyte A (1:4) (total volume: 1060 mL). Mannitol (3.26 g), potassium chloride (K, 26 mEq), magnesium sulphate (Mg, 2 g), lidocaine (130 mg), and sodium bicarbonate (13 mEq) were added to the del Nido solution. BC solution consists of mixed blood and Ringer's lactate (1:4) (total volume: 550 mL). Mannitol (10 g), potassium chloride (K, 46 mEq), magnesium sulphate (2.5 g), lidocaine (40 mg), and sodium bicarbonate (1 mEq) were added to the BC solution.

### Surgical Procedure

All subjects underwent a full sternotomy for surgical access under transoesophageal echocardiogram guidance. Cardioplegia solution was delivered by antegrade cardioplegia cannula. The velocity was 200 mL/min and the solution's temperature was between 8-14 C degrees. The BC (1000 mL) solution was administered antegradely every 15 to 20 minutes. A single dose (1000 mL) of the del Nido solution was administered antegradely. After the surgeon completed the distal coronary anastomoses, a left atriotomy was performed to expose and excise the mitral valve. The annulus of the mitral valve was sized, and the prosthesis was sutured to the annulus with 25 coated, braided polyester stitches. The correct positioning was confirmed and the left atrium was closed in the standard fashion. The de novo mitral valve competency was assessed using transoesophageal echocardiography. Once the competency of the valve was confirmed, the cross-clamp was removed. A proximal coronary anastomosis was performed with a side clamp. After the surgery, the subjects were transferred to the intensive care unit and they were extubated 6-8 hours postoperatively. None of the subjects with preoperative atrial fibrillation underwent a maze procedure.

The intraoperative parameters were recorded, including aortic clamp time, CPB time, cardioplegia volume, bypass graft number, use of intraoperative defibrillation, and use of inotropic support (adrenalin/other).

Creatine kinase-MB (CK-MB) and troponin T, blood count (fresh frozen plasma [FFP], red blood cells [RBCs], and thrombocytes), and blood creatinine levels were assessed at 1, 6, 12, and 24 hours postoperatively. Haemoglobin (Hgb), haematocrit (Htc), glucose, and K levels were assessed at 6 and 12 hours postoperatively.

Complications such as myocardial infarction, acute renal insufficiency, atrial fibrillation, other arrhythmias, respiratory insufficiency, transient ischemic attack (TIA)/stroke, the need for a pacemaker, reoperation due to haemorrhage, infection, and death were noted.

The length of total intensive care and hospital stays was noted. All subjects underwent an echocardiographic assessment for EF and valve function before discharge from the hospital.

### Statistical Analysis

The Number Cruncher Statistical System (NCSS) 2007 (Kaysville, Utah, USA) was used for statistical analysis. Descriptive statistical methods (mean, standard deviation [SD], median, frequency, ratio, minimum, and maximum) were used. Quantitative data with normal distribution were compared using Student's t-test, and the comparison of quantitative data without normal distribution was performed using Mann-Whitney U test. Comparisons in groups for data with normal distribution were performed using paired-samples t-test, and comparisons in groups for data without normal distribution were performed using Wilcoxon sign test. For qualitative data, Pearson's chi-square test, Fisher's exact test, and Fisher-Freeman-Halton test were used. A *P*-value less than 0.05 was considered significant.

## RESULTS

For the study period, 30 subjects met the inclusion criteria for the DNC group (combined CABG [≤ 3 bypass grafts] and mitral stenosis surgery). The data were compared with 30 subjects who had previously received BC by the same surgeon and met the inclusion criteria. Twenty-two subjects were excluded from the study.

The subjects' baseline characteristics are presented in Table 1. The mean age of DNC and BC groups was 69.53±6.73 and 67.63±5.56 years, respectively, (*P*=0.272). The subjects did not differ in terms of sex distribution (*P*=0.432), NYHA functional status (*P*=1.000), or cardiac and non-cardiac comorbidities. The groups also did not differ in preoperative echocardiographic parameters. The preoperative EF (%) of DNC and BC groups was 48.63±5.61 and 47.20±7.36%, respectively, (*P*=0.400). The preoperative valvular area in DNC and BC groups was 1.06±0.24 and 1.11±0.23 cm^2^, respectively, (*P*=0.376). And the preoperative gradient (mmHg) for DNC and BC groups was 9.33±1.37 and 8.93±1.29, respectively, (*P*=0.249).

**Table 1:** Subjects baseline characteristics according to cardioplegia type.

		Cardioplegia type		*P*
		Del Nido (n=30)	Blood (n=30)	
Age	Min-max (median)	54-81 (70)	54-80 (67)	a0.272
	Mean±SD	69.53±6.73	67.63±5.56	
Gender	Female	11 (36.7)	14 (46.7)	b0.432
	Male	19 (63.3)	16 (53.3)	
NYHA functional status	Class 1	2 (6.7)	2 (6.7)	c1.000
	Class 2	11 (36.7)	10 (33.3)	
	Class 3	15 (50)	15 (50)	
	Class 4	2 (6.7)	3 (100)	
Cardiac comorbidity				
Hypertension		23 (76.7)	22 (73.3)	b0.766
Dyslipidemia		18 (60)	16 (53.3)	b0.602
Rhythm-atrial fibrillation		8 (26.7)	9 (30)	b0.774
Mitral valve insufficiency		5 (16.7)	4 (13.3)	d1.000
Non-cardiac comorbidity				
Tobacco use		15 (50)	17 (56.7)	b0.605
Alcohol consumption		4 (13.3)	3 (10)	d1.000
Diabetes mellitus		16 (53.3)	12 (40)	b0.301
Chronic obstructive pulmonary disease		7 (23.3)	5 (16.7)	b0.519
Chronic renal insufficiency		3 (10)	2 (6.7)	d1,000
Preoperative TIA or stroke		2 (6.7)	3 (10)	d1,000
Preoperative echocardiographic parameters				
Preoperative ejection fraction (%)	Min-max (median)	35-60 (49)	30-55 (49)	a0.400
	Mean±SD	48.63±5.61	47.20±7.36	
Preoperative valvular area (cm^2^)	Min-max (median)	0,7-1,5 (1,1)	0.7-1.5 (1.1)	a0.376
	Mean±SD	1.06±0.24	1.11±0.23	
Preoperative gradient (mm/HG)	Min-max (median)	7-12 (9)	7-12 (9)	a0.249
	Mean±SD	9.33±1.37	8.93±1.29	

a Student-t test;

b Pearson chi-square test;

c Fisher-Freeman-Halton test;

d Fisher's exact test.

NYHA=New York Heart Association; SD=standard deviation; TIA=transient ischemic attack

**Fig. 1:**
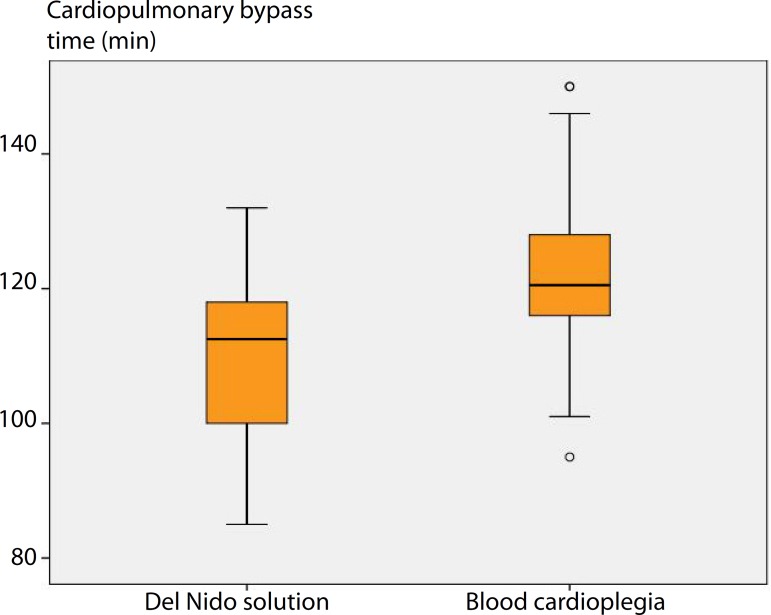
Comparison of cardiopulmonary bypass time (min) between del Nido cardioplegia and blood cardioplegia.

**Fig. 2:**
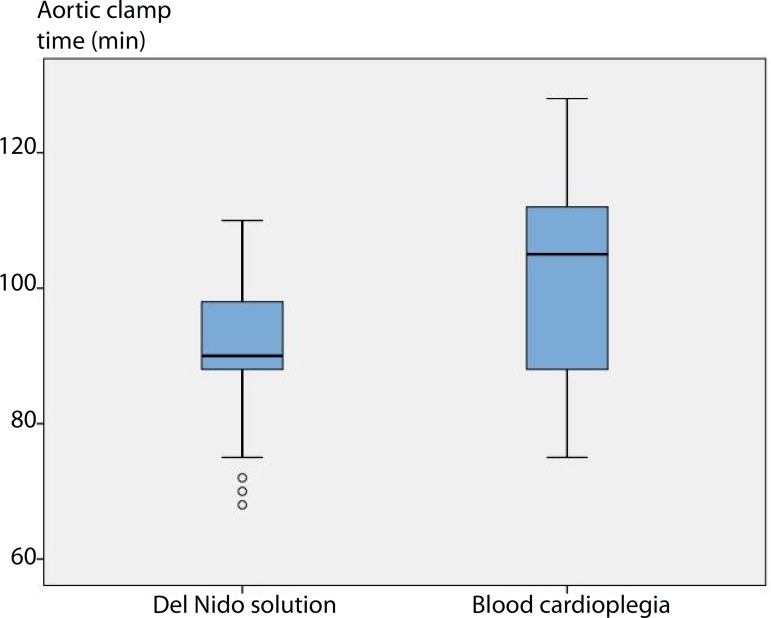
Comparison of aortic clamp time (min) between del Nido cardioplegia and blood cardioplegia.

The intraoperative data are presented in Table 2. The number of bypass grafts and mitral valve replacement types did not differ between the groups (*P*=0.927 and *P*=0.100, respectively). Compared with the BC group, the DNC group demonstrated significantly lower cardioplegia volume (BC = 1130.00±194.1 mL and DNC = 884.33±156.8 mL, *P*=0.001), CPB time (DNC = 110.90±12.52 min and BC = 121.70±13.57 min, *P*=0.002) (Figure 1), aortic clamp time (DNC = 91.37±11.58 min and BC = 101.37±13.87 min, *P*=0.004) (Figure 2), and number of intraoperative defibrillation procedures (DNC = 6 cases and BC = 21 cases, *P*=0.001).

**Table 2:** Intraoperative data according to cardioplegia type.

		Cardioplegia type		*P*
		Del Nido (n=30)	Blood (n=30)	
No. bypass graft	1	3 (10)	3 (10)	c0.927
	2	14 (46.7)	16 (53.3)	
	3	13 (43.3)	11 (36.7)	
Cardioplegia volume (mL)	Min-max (median)	700-1350 (930)	1000-1500 (1000)	e0.001**
	Mean±SD	884.33±156.8	1130.00±194.1	
Cardiopulmonary bypass time (min)	Min-max (median)	85-132 (112.5)	95-150 (120.5)	a0.002**
	Mean±SD	110.90±12.52	121.70±13.57	
Aortic clamp time (min)	Min-max (median)	68-110 (90)	75-128 (105)	a0.004**
	Mean±SD	91.37±11.58	101.37±13.87	
Intraoperative defibrillation		6 (20)	21 (70)	b0.001**
Mitral valve replacement type	Mechanic valve	7 (23.3)	13 (43.3)	b0.100
	Bioprosthetic valve	23 (76.7	17 (56.7)	

a Student-t test;

b Pearson chi-square test;

c Fisher-Freeman-Halton test;

e Mann-Whitney U test

** *P*<0.01; SD=standard deviation

After surgery, the subjects were transferred to the intensive care unit. Biochemical parameters for the first postoperative day are presented in Table 3. The CK-MB levels at 1 and 24 hours postoperatively were significantly lower in the DNC group (DNC = 5.82±4.72 ng/mL and BC = 7.27±4.69 ng/mL, *P*=0.041, and DNC = 13.30±7.72 ng/mL and BC = 17.53±7.26 ng/mL, respectively, *P*=0.001). The troponin levels at 1 hour did not differ between the groups (DNC = 0.13±0.05 ng/mL and BC = 0.24±0.25 ng/mL, *P*=^e^**0.**099). The troponin levels at 24 hours postoperatively were significantly lower in the DNC group (DNC = 0.28±0.41 ng/mL and BC = 0.67±1.03 ng/mL, *P*=0.001). The Hgb levels at 6 and 24 hours postoperatively were significantly higher in the DNC group than in the BC group (10.99±1.81 g/dL *vs*. 9.80±1.28 g/dL, *P*=0.005, and 10.86±1.11 g/dL *vs*. 9.94±0.79 g/dL, *P*=^a^**0.**001, respectively). The postoperative Htc levels at 6 and 24 hours were significantly higher in the DNC group than in the BC group (32.43±4.94 *vs*.29.10±3.75, *P*=^a^0.005, and 32.33±3.02 *vs*. 29.60±2.36, *P*=0.001, respectively). The postoperative K levels were lower in the DNC group than in the BC group at the first postoperative hour (4.09±0.82 mM *vs*. 4.40±0.83 mM, respectively, *P*=0.031), whereas the K levels at 24 hours postoperatively did not differ between the groups (*P*=0.223). There was no significant difference between the groups for inotropic support regardless of the inotropic agent used (*e.g*., epinephrine or other inotropic agent) (*P*=1.000 and *P*=0.542, respectively). The postoperative creatinine levels at 24 hours (*P*=^e^**0.**847) and postoperative glucose levels at 6 and 24 hours (*P*=0.564 and *P*=0.604, respectively) did not differ between the groups.

**Table 3:** Postoperative biochemical parameters according to cardioplegia type.

	Cardioplegia type		
	Del Nido (n=30)	Blood (n=30)	*P*
Inotropic support in first 24 hours	Norepinephrine (n)	3 (10)	d1.000
	Other inotropes (n)	6 (20)	b0.542
CK-MB (ng/mL), 1^st^ hour	Min-max (median)	2.4-25 (5.8)	e0.041*
	Mean±SD	7.27±4.69	
CK-MB (ng/mL), 24^th^ hour	Min-max (median)	9.1-50 (17.4)	e0.001**
	Mean±SD	17.53±7.26	
	**Difference**	10.27±5.14	e0.004**
	*P*	f0.001**	
Troponin T (ng/mL), 1^st^ hour	Min-max (median)	0.05-1.2 (0.16)	e0.099
	Mean±SD	0.24±0.25	
Troponin T (ng/mL), 24^th^ hour	Min-max (median)	0.1-6 (0.5)	e0.001**
	Mean±SD	0.67±1.03	
	**Difference**	0.44±0.87	e0.001**
	*P*	f0.001**	
Haemoglobin (g/dL), 6^th^ hour	Min-max (median)	7.9-12.1 (10)	a0.005**
	Mean±SD	9.80±1.28	
Haemoglobin (g/dL), 24^th^ hour	Min-max (median)	8.7-11.7 (9.9)	a0.001**
	Mean±SD	9.94±0.79	
	**Difference**	0.13±1.22	e0.387
	*P*	g0.554	
Haematocrit, 6^th^ hour	Min-max (median)	24-36 (29)	a0.005**
	Mean±SD	29.10±3.75	
Haematocrit, 24^th^ hour	Min-max (median)	26-35 (29)	a0.001**
	Mean±SD	29.60±2.36	
	**Difference**	0.50±3.60	e0.457
	*P*	g0.453	
Postoperative 24^th^ hour creatinine (mg/dL)	Min-max (median)	0.7-5.1 (1.05)	e0.847
	Mean±SD	1.44±1.15	
K^+^ (mM), 1^st^ hour	Min-max (median)	3.5-6.3 (4.1)	e0.031*
	Mean±SD	4.40±0.83	
K^+^ (mM), 24^th^ hour	Min-max (median)	3.4-6.4 (4.1)	e0.223
	Mean±SD	4.47±0.89	
	**Difference**	0.06±0.47	e0.132
	*P*	f0.591	
Glucose (mg/dL), 6^th^ hour	Min-max (median)	85-296 (128)	e0.564
	Mean±SD	164.00±70.23	
Glucose (mg/dL), 24^th^ hour	Min-max (median)	89-350 (125)	e0.604
	Mean±SD	176.60±81.23	
	**Difference**	12.60±33.65	e0.876
	*P*	f0.115	

a Student-t test;

b Pearson chi-square test;

d Fisher's Exact test;

e Mann-Whitney U test;

f Wilcoxon Signed Ranks

g Paired Samples Test;

* *P*<0.05;

** *P*<0.01.

CK-MB=creatine kinase-MB; SD=standard deviation

The intensive care unit intubation period (hours) was significantly lower in the BC group (DNC = 8.13±12.21 and BC = 6.82±1.57, *P*=0.037); however, the intensive care unit stay (days) was not different between the groups (*P*=0.163) (Table 4).

**Table 4:** Postoperative data according to cardioplegia type.

	Cardioplegia type		
	Del Nido (n=30)	Blood (n=30)	*P*
Postoperative red blood cell transfusion (n)	None	18 (60)	c0.754
	1 unit	7 (23.3)	
	2 units	2 (6.7)	
	3 units	3 (10)	
Postoperative plasma transfusion (n)	None	16 (53.3)	c0.001**
	1 unit	7 (23.3)	
	2 units	3 (10)	
	3 units	4 (13.3)	
Postoperative platelet transfusion (n)	None	27 (90)	c0.611
	1 unit	0 (0.0)	
	2 units	0 (0.0)	
	3 units	3 (10)	
Complications	Low cardiac output syndrome	3 (10)	d1.000
	Myocardial infarction	1 (3.3)	d1.000
	Acute renal insufficiency	3 (10)	d1.000
	Atrial fibrillation	8 (26.7)	b0.095
	Respiratory failure	4 (13.3)	d0.671
	Stroke/TIA	1 (3.3)	d1.000
	Permanent pacemaker	0 (0.0)	-
	Reoperation for bleeding	3 (10)	d1.000
	Infection	1 (3.3)	d1.000
	Hospital mortality	1 (3.3)	d1.000
Intensive care unit intubation period (hours)	Min-max (median)	5-11 (6.5)	e0.037*
	Mean±SD	6.82±1.57	
Intensive care unit stay (days)	Min-max (median)	2-10 (2)	e0.163
	Mean±SD	2.53±1.57	
Hospital stay (days)	Min-max (median)	5-10 (6)	e0.142
	Mean±SD	6.30±1.58	
Pre-discharge ejection fraction (%)	Min-max (median)	30-55 (45)	e0.005**
	Mean±SD	45.72±5.86	

b Pearson chi-square test;

c Fisher Freeman Halton Test;

d Fisher's Exact test;

e Mann-Whitney U test;

* *P*<0.05;

** *P*<0.01.

SD=standard deviation; TIA=transient ischemic attack

The need for postoperative transfusion of RBCs or platelets did not differ between the groups (*P*=0.754 and *P*=0.611, respectively). The postoperative plasma transfusion rate was significantly higher in the DNC group than in the BC group (*P*=0.001). Regarding complications, 17 events occurred in the DNC group and 25 events occurred in the BC group. All the comparisons for postoperative complications were not statistically significant.

At pre-discharge echocardiography, the DNC group demonstrated a significantly better EF percentage than the BC group (47.79±5.50 *vs*. 45.72±5.86, respectively, *P*=0.005). The length of hospital stay was not different between the groups (*P*=0.142).

## DISCUSSION

During heart surgery, myocardial protection is an important consideration. Several cardioplegia solutions are available; however, there is no consensus concerning the optimal composition or technique^[4]^. The current study compared the safety and efficacy of DNC and BC in 30 matched subjects. Compared with the BC group, the DNC group demonstrated better intraoperative parameters, including lower cardioplegia volume, CPB time, aortic clamp time, and need for intraoperative defibrillation.

Common cardioplegia techniques include BC, histidine-tryptophan-ketoglutarate solution, and DNC^[1]^. The del Nido solution contains Plasma-Lyte A and a crystalloid component. The base solution has the same electrolyte composition as extracellular fluid, and the crystalloid component contains mannitol, magnesium sulphate, sodium bicarbonate, potassium chloride, and lidocaine^[5]^. Lidocaine (Na^+^ channel blocker) and Mg^2+^ (Ca^2+^ competing agent/Ca^2+^ channel blocker) decrease intracellular Ca^2+^ concentration, myocardial excitability, cellular metabolism, and energy consumption^[7,8]^. Although originally designed for a child's immature heart, the del Nido solution is offered as a new alternative to protect the ischaemic myocardium^[6]^.

Available literature did not demonstrate homogeneous data regarding DNC. However, initial experiences demonstrated its safety in CABG and isolated or combined valve surgery^[1]^. The use of DNC in CABG has been addressed in a few studies. Guajardo Salinas et al.^[9]^ compared DNC (n=134) with BC (n=230). Except for the mean cardioplegia volume, mean number of cardioplegia doses, and defibrillation after cross-clamp removal, the groups demonstrated similar intraoperative and postoperative properties. The use of DNC resulted in a lower need for defibrillation in the subjects who underwent CABG. An equivalent efficiency of DNC was previously demonstrated by Timek et al.^[10]^, who reported the results for 100 propensity-score-matched subjects who underwent CABG. The use of DNC resulted in lower glucose levels than those for BC. In a previous report, Yerebakan et al.^[11]^ demonstrated the safety of DNC in high-risk CABG surgery after acute myocardial infarction. Of 48 subjects who received DNC, the authors reported significantly shorter mean CPB and cross-clamp times; however, other intraoperative and postoperative data were similar between the groups.

Among the studies that addressed the use of DNC in mitral valve surgery, Yammine et al.^[7]^ compared modified DNC with whole BC in 79 matched subjects. The included subjects underwent valve procedures and/or CABG or mechanical valve implant. The postoperative 24-hour CK-MB levels were high in the DNC group. Except for the CK-MB levels, the operative parameters and postoperative comparisons were similar in both groups. Kim et al.^[12]^ compared the use of DNC with BC in 39 matched subjects. Although most of the subjects underwent isolated valvular surgery, other subjects who underwent aortic replacement surgery, CABG surgery, or had a congenital heart disease or tumour were also enrolled in the study. They've found no association between peak troponin I levels and left ventricular mass/aortic clamp time between the groups. Mick et al.^[13]^ compared the use of DNC with Buckberg cardioplegia in isolated aortic (n=85) or mitral valve surgery (n=110). The results demonstrated the safety of DNC in adult subjects who underwent isolated valve surgery. In aortic valve surgery, the use of DNC significantly reduced aortic clamp time, bypass time, and operating room time; however, in mitral valve surgery, the use of DNC was found to only be advantageous in case of postoperative insulin need. In both groups, the postoperative troponin levels as well as the left ventricular EF were similar.

Aortic valve procedures were also addressed in studies. Sorabella et al.^[14]^ compared the use of DNC (n=52) with BC (n=65) in isolated aortic valve procedures. Except for total cardioplegia volume, no significant difference was found between the groups. Of 54 matched subjects, Ota et al.^[15]^ reported shorter CPB and cross-clamp times when DNC was used. Postoperative complications as well as length of intensive care and hospital stays did not differ between the groups. Vistarini et al.^[16]^ reported less atrial fibrillation, lower CK-MB levels, and lower insulin requirement associated with the use of DNC in minimally invasive aortic valve surgery. Hamad et al.^[2]^ evaluated the effect of DNC in CABG combined with aortic valve surgery in 25 subjects. CPB time, cross-clamp time, and postoperative CK-MB and troponin levels were lower in the surgeries with use of DNC.

The basic advantages of DNC are single-dose application and glucose-free ingredients^[6]^. DNC was administered as a single dose for subjects who underwent procedures of less than 90 minutes duration. The single-dose application decreased CPB and cross-clamp times. A recent meta-analysis involving 9 studies reported the effect of DNC in 1501 subjects (4 studies involved isolated valve procedures, 3 studies involved CABG procedures, and 2 studies reported valve procedures or CABG). The results of the meta-analysis reported shorter CPB and cross-clamp times when DNC was used^[1]^. Cardioplegia volume, blood glucose levels, ventilation time, and length of intensive care stay were also decreased for DNC when compared with those for BC. BC requires multiple interruptions, which is an additional factor for ischaemic damage. The single-dose application was associated with a lower cardioplegia volume and less haemodilution, which decreases the transfusion requirement according to literature^[1,2]^. In the present study, the need for RBCs and platelet transfusion rates were similar in DNC and BC groups. Although the postoperative plasma transfusion need was lower in the BC group, the importance of this finding could not be clearly described. Having glucose-free ingredients in the solution is important for subjects who have diabetes mellitus. However, no significant difference was observed between the groups in the present study.

The use of DNC demonstrated lower CK-MB and troponin levels, which are sensitive biomarkers for cardiac injury. The lower levels in our study were a sign of better myocardial protection. The DNC group also demonstrated higher Hgb and Htc levels; however, RBC transfusion rates were similar for both groups. In the present study, the use of DNC demonstrated similar postoperative complication rates and intensive care and hospital stay rates. Although the BC group demonstrated significantly shorter intensive care unit intubation period than the DNC group, this difference was not regarded as clinically significant. Before discharge, the subjects underwent an echocardiographic assessment. A higher EF rate in the DNC group was also a sign of better myocardial protection.

This study presents a single-center, single-surgeon experience in a retrospective design, and there are limitations related to such design. Long-term follow-up was not conducted, which made it difficult to interpret some findings such as lower cardiac marker levels and better postoperative EF rates. As an initial experience, our data added observational value, but there is a need for randomised, multicenter trials comparing different solutions in different cardiac procedures.

## CONCLUSION

The current study results showed better intraoperative and postoperative chemical parameters when DNC was used. The need for a lower cardioplegia volume and an uninterrupted procedure are the main advantages of DNC. DNC is at least equivalent to BC and it is a safe alternative to BC in CABG combined with mitral valve surgery in adults.

**Table:** 

Authors' roles & responsibilities	
AAK	Substantial contributions to the conception or design of the work; or the acquisition, analysis, or interpretation of data for the work; drafting the work or revising it critically for important intellectual content; final approval of the version to be published
ST	Substantial contributions to the conception or design of the work; or the acquisition, analysis, or interpretation of data for the work; drafting the work or revising it critically for important intellectual content; final approval of the version to be published; agreement to be accountable for all aspects of the work in ensuring that questions related to the accuracy or integrity of any part of the work are appropriately investigated and resolved
